# Management of lethal recessive alleles in beef cattle through the use of mate selection software

**DOI:** 10.1186/s12711-019-0477-3

**Published:** 2019-08-06

**Authors:** Lindsay R. Upperman, Brian P. Kinghorn, Michael D. MacNeil, Alison L. Van Eenennaam

**Affiliations:** 1 Department of Animal Science, University of California, Davis, CA 95616 USA; 2 School of Environmental and Rural Science, University of New England, Armidale, NSW 2351 Australia; 3 Delta G, Miles City, MT 59301 USA; 4 University of the Free State, Bloemfontein, 9300 South Africa; 5 Present Address: Department of Animal Science, University of Nebraska, Lincoln, NE 68583 USA

## Abstract

**Background:**

Recessive loss-of-function (LOF) alleles at genes which are essential for life, can result in early embryonic mortality. Cattle producers can use the LOF carrier status of individual animals to make selection and mate allocation decisions.

**Methods:**

Two beef cattle breeding strategies i.e. (1) selection against LOF carriers as parents and (2) simultaneous selection and mate allocation to avoid the occurrence of homozygous offspring in three scenarios, which differed in number and frequency of LOF alleles were evaluated using the mate selection program, MateSel. Scenarios included (a) seven loci with high-frequency LOF alleles, (b) 76 loci with low-frequency LOF alleles, and (c) 50 loci with random high- and low-frequency LOF alleles. In addition, any savings resulting from the information obtained by varying the percentage (0–100%) of the herd genotyped, together with segregation analysis to cover ungenotyped animals, were calculated to determine (1) which percentage optimized net profit for a fixed cost of genotyping ($30/test), and (2) the breakeven cost for genotyping.

**Results:**

With full knowledge of the LOF alleles carried by selection candidates, the most profitable breeding strategy was always simultaneous selection and mate allocation to avoid homozygous affected offspring (*aa*) as compared to indiscriminate selection against carrier parents (*Aa*). The breakeven value of genotyping depended on the number of loci modeled, the LOF allele frequencies, and the mating/selection strategies used. Genotyping was most valuable when it was used to avoid otherwise high levels of embryonic mortalities. As the number of essential loci with LOF alleles increased, especially when some were present at relatively high minor allele frequencies, embryonic losses increased, and profit was maximized by genotyping 10 to 20% of a herd and using that information to reduce these losses.

**Conclusions:**

Genotyping 100% of the herd was never the most profitable outcome in any scenario; however, genotyping some proportion of the herd, together with segregation analysis to cover ungenotyped animals, maximized overall profit in scenarios with large numbers of loci with LOF alleles. As more LOF alleles are identified, such a mate selection software will likely be required to optimally select and allocate matings to balance the rate of genetic gain, embryonic losses, and inbreeding.

## Background

For commercial beef cow-calf operators, fertility is the most important economic trait among the breeding objectives and outweighs even growth and carcass traits [1, 2]. Beef cattle fertilization rates to a single artificial insemination (AI) service of about 90 to 100% have been observed, and yet the subsequent calving rates reach about 55%, which suggests that at least 35% of pregnancies are lost between fertilization and calving [3]. The low frequencies of recessive loss-of-function (LOF) alleles of genes that are essential for life may be associated with part of this early embryonic mortality. Genomic tools have enabled the identification of early embryonic mortality LOF mutations in dairy cattle that are evidenced by decreased fertility scores in genetic evaluations [4]. Recent studies have revealed additional lethal LOF alleles in beef and dairy cattle populations, and at least 17 haplotypes have been identified [5], with carrier frequencies ranging from 2.2 to 23.4%, and recessive haplotype frequencies ranging from 0.1 to 15.2% [5–8] (Table 1).

**Table 1:** Reported allele frequencies for recessive haplotypes in both dairy and beef cattle

Breed	Haplotype frequency (%)	Carrier frequency (%)	Functional/gene name	References
Ayrshire	13.0		PIRM/UBE3B	[9, 10]
		20	RPAP2	[11]
Belgian blue		10	MYH6	[7]
		10.2	SNAPC4	[7]
		3.8	RPIA	[7]
		2.6	EXOSC4	[7]
		2.2	MED22	[7]
Braunvieh		14		[12]
		13 (3.4)	TUBD1	[13]
Fleckvieh		2.9		[14]
		4.1	SLC2A2	[14]
		3.3		[14]
		3.3	SUGT1	[14]
Brown Swiss	6.67		–	[4]
	7.78		*TUBD1*	[13]
	2.19		SDM/*SPAST*	[15, 16]
	3.61		SMA/KDSR(*FVT1*)	[17, 18]
	1.56		Weaver/*PNPLA8*	[19, 20]
Holstein	2.76		Brachyspina/*FANCI*	[21, 22]
	1.92	4.5	*APAF1*	[23, 24]
	1.66		–	[4, 25]
	2.95	4.7	*SMC2*	[25, 26]
	0.37		*GART*	[24]
	2.22	5.5	*TFB1M*	[9, 27]
	0.25		BLAD/*ITGB2*	[28]
	1.37	8	CVM/*SLC35A3*	[29, 30]
	0.1		DUMPS/*UMPS*	[31]
Jersey	12.1	23.4	CWC15	[32]
	1.3		–	[33]
		13.2	OBFC1	[7]
Montbéliarde		18	PFAS	[34, 35]
		14	SLC37A2	[35]
Normande		3.8		[35]
Japanese black		4.8	ANXA10	[36]
Angus	7.8		GEMIN2	[6]
	15.2		ZFAT	[37]
Charolais	14.4		–	[37]
Simmental	8.8		–	[37]

Modified from Cole et al. [8] and Georges et al. [5]. Reproduced with permission via Copyright Clearance Center

Genotyping provides an opportunity to evaluate the LOF allele status for individual animals, and this information could be used to avoid matings that result in early embryonic losses due to the occurrence of homozygous LOF offspring. However, as research identifies an ever-increasing number of essential loci that potentially harbor LOF alleles, computerized decision support programs will likely be needed to make optimal mating decisions given the computational complexities associated with the incorporation of LOF information from multiple essential gene loci into genetic improvement programs [38].

Mate allocation, which is the process of selecting mating pairs from a population of females and some portfolio of males, can be used to help avoid pairing animals that carry LOF alleles at the same locus. A constrained mate selection algorithm, MateSel, was developed to optimize mate selection decisions—to simultaneously optimize both mate selection and mate allocation decisions [39]. For example, the program can maximize the rate of genetic gain towards a given breeding objective under the constraint of holding the inbreeding rate to a user-defined level, plus other logistical constraints required by breeders. It allows breeders to simulate mate selection decisions and interactively constrain different variables to observe the impact of such constraints on the rate of genetic progress and other predicted outcomes. Thus, MateSel offers the opportunity to compare and contrast different approaches to the management of recessive conditions.

The purpose of this study was to test the effectiveness of both mate selection for reducing the detrimental impact of LOF loci on herd fertility, and segregation analysis for reducing the genotyping costs involved. Three scenarios with different numbers of essential loci and LOF allele frequencies were set up to compare two breeding strategies for selecting against recessive lethal alleles. The first strategy was (1) selection against LOF carriers as parents, and the second was (2) simultaneous mating and selection against the occurrence of homozygous offspring (i.e., matings between carriers at the same locus). This was carried out within a given population using a set of constraints, selection indices, and different proportions of whole herd genotyping (0–100%). The weighting that maximized profit per mating ($P) from each mating strategy given perfect knowledge of genotypes was determined as described in Upperman et al. [40], and then the effect of genotyping varying percentages, from 0 to 100%, of the herd was examined. The percentage of herd genotyped that optimized net profit for a fixed cost of genotyping ($30/test) was determined, as well as the breakeven genotyping cost at which the expenses associated with genotyping different percentages of the herd equaled the value derived from using that genotyping information to avoid embryonic losses for the three scenarios.

## Methods

### Modeling of the dataset

PopSNP (version 1.6), a software program that populates single nucleotide polymorphisms (SNPs) into a given pedigree dataset according to Mendelian segregation laws [41], was used to create three scenarios that included varying numbers of essential loci and LOF allele frequencies. Scenario (A) included seven loci with LOF alleles at high frequencies (mean frequency 0.0847 ranging from 0.0527 to 0.1001), scenario (B) included 76 loci with LOF alleles at low frequencies (mean frequency 0.0112 ranging from 0.0004 to 0.0695), and scenario (C) included 50 loci with LOF alleles with random high and low frequencies (mean frequency 0.0488 ranging from 0.0044 to 0.1436) (Table 2).

**Table 2:** Allele frequencies for the three scenarios with different numbers of loci

Scenario	Number of loci	Mean frequency	Standard deviation	Minimum frequency	Maximum frequency
A	7	0.0847	0.0151	0.0527	0.1001
B	76	0.0112	0.0125	0.0004	0.0695
C	50	0.0488	0.0307	0.0044	0.1436

Scenario A uses high-frequency loss-of-function alleles at seven essential loci, scenario B uses low-frequency loss-of-function alleles at 76 essential loci, and scenario C uses both high- and low-frequency loss-of-function alleles at 50 essential loci

For scenario (B), 83 loci with LOF allele frequencies higher than 0 were simulated using PopSNP. Setting the cut-off value for allele frequency at 0.07 and higher eliminated seven alleles, leaving a total of 76 “low” frequency variants in scenario (B).

The simulated LOF SNPs were populated into an Angus pedigree dataset provided by Mike Kasten with 85 male candidates, 169 female candidates, and 546 ancestors following a burn-in of 1000 generations to create a resource for populating foundation animals with initial genotypes. A genome size of 3 Gb across 29 chromosomes was modeled based on the size and chromosome complement of the bovine genome [42]. The Kosambi mapping function was used to calculate recombination fractions. A mutation rate of 2.2 × 10^−9^ was used to calculate generation and population size parameters [43]. Any candidates or ancestors that would have had a homozygous recessive lethal genotype (*aa*) were assumed dead and not allowed in the population. The dataset included American Angus Association’s expected progeny differences (EPD) and economic selection index values for each candidate. If EPD values were not reported for an animal, average values of the breed database were used.

### Maternal economic selection index

Ochsner et al. [44, 45] outlined procedures on how to develop a maternal economic selection index ($M) given EPD information. These procedures were followed based on selection objective weightings described by MacNeil [46]. These included heifer pregnancy, calving ease direct, calving ease maternal, weaning weight direct, weaning weight maternal (milk), and stayability. Selection criteria included heifer pregnancy, calving ease direct, calving ease maternal, weaning weight direct, weaning weight maternal (milk), and back fat thickness. Due to incomplete parameter estimations for the simulated dataset, estimates of heritability, genotypic variance, phenotypic variance, and genetic correlation were based on estimates in the literature that originated mainly from reference Angus or British breed populations [47–52] (Table 3).

**Table 3:** Genetic parameters with estimated heritabilities on the diagonal (in italics) and genetic correlations above the diagonal

	HP	CED	CEM	WWD	WWM	FT	STAY	GLS^a^
HP	*0.21*	0	0	0.29	0	0	0	0
CED		*0.2*	− 0.3	− 0.16	0	0	0	0
CEM			*0.1*	0	0	0	0.25	0
WWD				*0.2*	− 0.17	0.1	0	0
WWM					*0.14*	0	− 0.15	0
FT						*0.4*	0.53	0
STAY							*0.15*	0
GLS								*0*

*HP* heifer pregnancy, *CED* calving ease direct, *CEM* calving ease maternal, *WWD* weaning weight direct, *WWM* weaning weight maternal or milk, *FT* fat thickness, *STAY* stayability, *GLS* genetic load score (this reflects long-term impact on expression of LOF mortality; see text for details)

^a^Assuming total independence of GLS

An additional metric was developed to represent the number of recessive LOF alleles carried by each sire, referred to as the genetic load score (GLS). For each sire, the square of the minor allele frequency (q^2^) at each LOF locus that was heterozygous were summed. The resulting values were divided by the number of matings (100) within each run and weighted based on the number of matings allocated to each sire. This approach was chosen to reflect the long-term impact of the current selection of sires on expression of LOF mortality.

### Mate selection

MateSel (FortranDLL version 9.4) is a software program for tactical implementation of breeding programs based on an evolutionary algorithm [53]. It optimizes mate selection and allocation among a given group of females and males to maximize the rate of genetic gain towards a selection objective, while constraining parental coancestry for controlling long-term inbreeding to maintain genetic variation for future improvement. The resulting mating list accommodates optimal contributions of parents to future generations, together with other factors such as progeny inbreeding, practical constraints, and management of the allele/genotype frequencies for nominated markers. Two strategies can be applied to select against multiple recessive lethal alleles. The first strategy is selection against LOF carriers as parents, and the second is simultaneous mating and selection against the occurrence of homozygous offspring (i.e., matings between carriers at the same locus). To compare these strategies, for selecting against recessive LOF alleles or LOF genotypes, two parameters LethalA and LethalG (see page 20 of [53]) were added to MateSel [54]. LethalA is the probability of mortality in *grandprogeny* due to LOF loci, given random mating of progeny and using current candidate frequencies for the LOF loci. Selecting against LethalA discriminates against the assignment of matings to animals that carry lethal recessive alleles, irrespective of the mates allocated, and essentially targets long-term reduction in mortality. LethalG is the probability of mortality in ***progeny*** due to LOF loci. Mate selection against LethalG effectively selects against the occurrence of lethal homozygous genotypes (*aa*) resulting from carrier matings, and essentially targets short-term reduction in mortality. This allows for the use of carrier sires provided that they are not mated to females that are LOF carriers at the same essential loci [39].

These definitions of LethalA and LethalG differ from those of Van Eenennaam and Kinghorn [54], which related to numbers of LOF alleles and genotypes, rather than probabilities of mortality. The current definitions were used because of their more direct link to utility and simpler evaluation of outcomes in relation to other component objectives.

### Simulation parameters

**Fig. 1:**
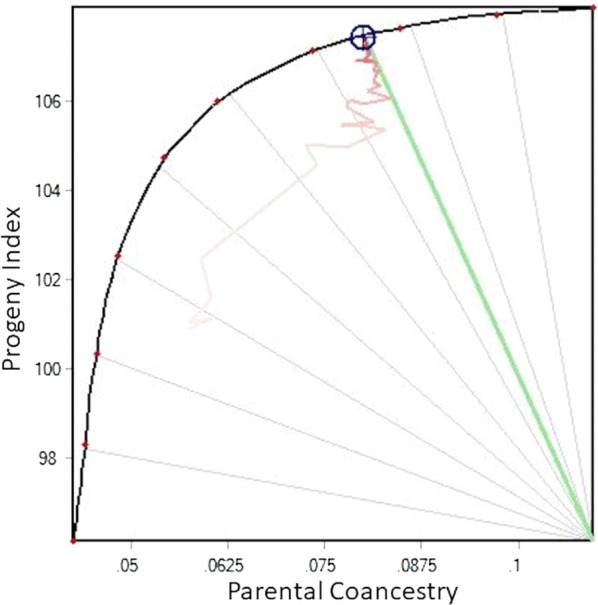
An example frontier response surface involving progeny index and parental coancestry. Reproduced from [39] with permission

Optimum mate selections were allocated for 100 matings, with progeny index ($M) as the key objective. No sire could be mated more than 50 times. A target compromise between genetic gain (as measured by the progeny index for $M) and inbreeding (as measured by parental coancestry) was set to 25 degrees in MateSel (see [39] for explanation). This target compromise is shown with the green line on Fig. 1.

This graph shows the balance between Progeny Index (Y axis), and Parental Coancestry (X axis). The black curved frontier shows the range of possible outcomes of optimal contributions (number of matings allocated to each candidate). The blue circle illustrates a solution for which a set of matings has been identified and which achieve the maximum rate of genetic change in progeny index given the constraint on parental coancestry set by the target compromise of 25 degrees (green line), where 0 degree corresponds to the maximum progeny index response and 90 degrees to minimum parental coancestry. The red trail shows the pathway that the evolutionary algorithm has made during convergence. Increasing the rate of genetic gain further would require to decrease the target compromise, thereby allowing selection of fewer and/or more related animals, thus accelerating the short-term genetic progress at the expense of long-term genetic variation.

Then, mate selection runs were performed with increasing weightings (0, 0.001, 0.01, 0.1, 1, 10, and 100) to both LethalA and LethalG mating strategies separately to decrease the predicted mortality in the long-term and short-term, respectively. In other words, we are saying how little (e.g. 0.001) or how much (e.g. 100) emphasis is placed on avoiding the use of carriers or recessive homozygous “dead” calves that show up within our calf crop. A cost of $200 was assigned to the occurrence of a homozygous, lethal “*aa*” genotype (embryonic mortality) [55, 56]. Profit per mating ($P) was calculated as $M (Index)—(LethalG × $200). In addition, the average sire index value ($MB) was calculated at each weighting. This value shows changes in the use of sires for the 100 matings, as well as the average sire GLS for each weighting.

### Percentage of herd genotyped

To model different percentages of herd genotyped, various values (0 = not genotyped and 1 = genotyped) were added to the dataset that started with a random assortment of 10% of the population (both sires and dams), which was initially denoted as genotyped. Those individuals then remained genotyped as the percentage of the herd genotyped increased, adding a random selection of the individuals (both sires and dams) that were genotyped for each 10% increase of the herd being genotyped. In all cases, ungenotyped animals had genotyped probabilities calculated by using Geneprob (version 3.3) [57], and these probabilities were used to help make mate selection decisions. A value of $30 was selected as the cost of genetic testing based on the current costs of beef cattle genetic tests ($29–$40) in the US market [58, 59]. Mate selections were then performed using the optimal weightings that maximized $P, under prevailing constraints, for each mating strategy in each of the three scenarios that had different numbers of loci and LOF allele frequencies (Table 2). Each scenario was replicated 100 times, each replicate with a different random sampling of true genotypes, for each percentage of the herd genotyped.

## Results

### Baseline parameters in the absence of herd genotyping

**Fig. 2:**
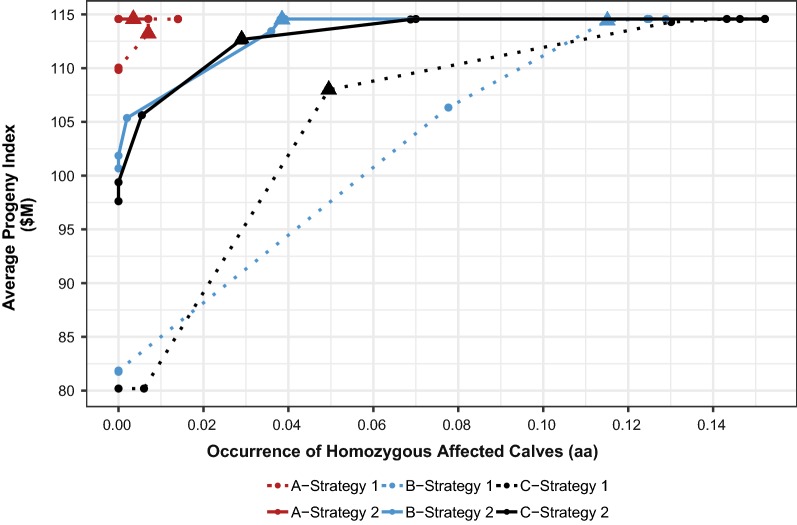
Average progeny index versus occurrence of affected calves per mating for three scenarios^a^ given two breeding strategies^b^. ^a^Scenario A (7 loci with high-frequency LOF alleles), scenario B (76 loci with low-frequency LOF alleles), and scenario C (50 with high- and low-frequency loci). ^b^Strategy 1: Selection against carrier parents with decreased weightings (L- > R: 100, 10, 1, 0.1, 0.01, 0.001, 0) against LethalA; Strategy 2: Selection and mate allocation to avoid homozygous offspring with decreased weightings (L- > R: 100, 10, 1, 0.1, 0.01, 0.001, 0) against LethalG. Filled triangle = profit maximizing weighting for a given scenario and breeding strategy

Figure 2 shows the average progeny index ($M) values versus the occurrence of affected calves per mating for the three scenarios with decreasing weightings (100, 10, 1, 0.1, 0.01, 0.001, 0) against LethalA or LethalG, and the profit maximizing weighting for each scenario and breeding strategy. Breeding strategy 1 (selection against carrier parents) to avoid embryonic lethality had little impact on average progeny index values and on the occurrence of homozygous affected calves when seven essential loci with LOF alleles were simulated since very few calves were lost in this scenario. However, in scenarios with 50 or more simulated loci, the occurrence of homozygous affected calves increased, and high weightings of LethalA in scenarios B and C resulted in a very large drop in average progeny index values. It should be noted that appropriate weightings under strategy 2 (selection against the occurrence of homozygous affected calves) can result in a considerable reduction of the occurrence of affected calves with very little compromise in average genetic gain.

First, we used MateSel to optimally select and allocate pairs for 100 matings without consideration of LOF alleles within the population for one generation. This is referenced as the “0% genotyping”, or base, run. The $P at 0% genotyping was $113.17, $89.67, and $84.14 for scenarios A, B, and C, respectively (Table 4).

**Table 4:** $P values for different percentages of herd genotyped in three scenarios^a^ given two selection strategies^b^

Level of genotyping	% Herd genotyping	$P					
		Strategy 1			Strategy 2		
		A	B	C	A	B	C
Base	0	$113.17	$89.67	$84.14	$113.17	$ 89.67	$84.14
Low	10	$113.17	$96.35	$87.91	$114.57	$ 98.24	$100.82
	20	$113.17	$99.71	$98.43	$114.57	$ 99.61	$102.57
	30	$113.17	$102.40	$97.54	$114.57	$101.62	$102.45
Intermediate	40	$113.17	$104.15	$99.01	$114.57	$103.19	$104.52
	50	$113.17	$104.47	$98.58	$114.57	$104.33	$106.33
	60	$113.17	$105.42	$99.02	$114.57	$105.39	$106.33
High	70	$113.87	$106.45	$98.27	$114.57	$106.39	$106.21
	80	$113.87	$106.55	$99.18	$114.57	$107.50	$107.59
	90	$113.87	$107.22	$99.06	$114.57	$108.12	$110.07
	100	$113.87	$107.35	$99.48	$114.57	$108.79	$110.31

^a^Scenario A (7 loci with high-frequency LOF alleles), scenario B (76 loci with low-frequency LOF alleles), and scenario C (50 with high- and low-frequency loci)

^b^Selection against carrier parents (strategy 1) and selection and mate allocation to avoid homozygous offspring (strategy 2)

At this base run, there was a loss of 0.7, 12.45, and 15.22 calves per 100 matings (Table 5), which equated to total herd losses of $140, $2490.43, and $3043.75 for scenarios A, B, and C, respectively, assuming an opportunity cost of $200 per embryo mortality.

**Table 5:** Predicted number of homozygous offspring (*aa*) with standard errors (SE) for different percentages of herd genotyped in three scenarios^a^ given two selection strategies^b^

Level of genotyping	% Herd genotyping	Predicted number of homozygous individuals per 100 matings (*aa*)					
		A		B		C	
Base	0	0.70 (0.000)		12.45 (0.000)		15.22 (0.000)	
	Strategy^b^	1	2	1	2	1	2
Low	10	0.70 (0.003)	0 (0.000)	9.11 (0.003)	8.17 (0.003)	12.42 (0.003)	6.77 (0.003)
	20	0.70 (0.003)	0 (0.000)	7.43 (0.003)	7.48 (0.002)	6.06 (0.003)	5.97 (0.003)
	30	0.70 (0.003)	0 (0.000)	6.08 (0.003)	6.47 (0.003)	6.05 (0.003)	5.92 (0.003)
Intermediate	40	0.70 (0.003)	0 (0.000)	5.21 (0.003)	5.69 (0.003)	4.80 (0.003)	4.87 (0.002)
	50	0.70 (0.003)	0 (0.000)	5.05 (0.003)	5.12 (0.002)	4.75 (0.003)	3.87 (0.003)
	60	0.70 (0.003)	0 (0.000)	4.58 (0.003)	4.59 (0.002)	4.7 5(0.003)	3.87 (0.003)
High	70	0.35 (0.003)	0 (0.000)	4.06 (0.003)	4.09 (0.002)	4.75 (0.003)	3.84 (0.003)
	80	0.35 (0.003)	0 (0.000)	4.01 (0.002)	3.53 (0.002)	4.35 (0.003)	3.23 (0.003)
	90	0.35 (0.002)	0 (0.000)	3.68 (0.003)	3.23 (0.002)	4.35 (0.002)	1.89 (0.001)
	100	0.35 (0.003)	0 (0.000)	3.61 (0.003)	2.89 (0.002)	4.25 (0.003)	1.83 (0.002)

^a^Scenario A (7 loci with high-frequency LOF alleles), scenario B (76 loci with low-frequency LOF alleles), and scenario C (50 with high- and low-frequency loci)

^b^Selection against carrier parents (strategy 1) and selection and mate allocation to avoid homozygous offspring (strategy 2)

These base prices show the impact that LOF alleles have on total profit for producers in the absence of herd genotyping in the three different scenarios. Per mating, this represents a loss of $1.40, $24.90, and $30.44 in $P for A, B, and C scenarios, respectively, relative to the theoretical maximum $P of $114.57, if there were no LOF alleles. Furthermore, $MB was $141.03 prior to consideration of lethal conditions, and the average GLS of the selected sires at this base level was 0.049, 0.287, and 0.333 for A, B and C scenarios, respectively (Table 6).

**Table 6:** Average sire index value ($MB) and genetic load scores (GLS) for different percentages of herd genotyped in three scenarios^a^ given two selection strategies^b^

Scenario^a^	Strategy^b^		Weightings against selection strategies						
			0	0.001	0.01	0.1	1	10	100
A	1	$MB	141.03	141.03	141.03	140.98	*140.61*	141.03	141.03
		GLS	0.049	0.027	0.027	0.024	*0.005*	0	0
	2	$MB	141.03	*141.03*	141.03	141.03	141.03	141.03	141.03
		GLS	0.049	*0.049*	0.049	0.049	0.049	0.049	0.049
B	1	$MB	141.03	141.03	140.97	*140.75*	130.57	86.74	86.50
		GLS	0.287	0.287	0.281	*0.265*	0.157	0	0
	2	$MB	141.03	*141.03*	141.03	141.03	132.21	118.97	115.34
		GLS	0.287	*0.284*	0.284	0.259	0.177	0.131	0.113
C	1	$MB	141.03	141.03	141.03	140.80	*133.75*	84.90	84.90
		GLS	0.333	0.333	0.332	0.298	*0.167*	0.012	0.012
	2	$MB	141.03	141.03	140.93	*138.81*	131.60	116.11	110.06
		GLS	0.333	0.333	0.333	*0.257*	0.163	0.100	0.080

In italic characters = the profit maximizing weighting for a given scenario and breeding strategy

^a^Scenario A (7 loci with high-frequency LOF alleles), scenario B (76 loci with low-frequency LOF alleles), and scenario C (50 with high- and low-frequency loci)

^b^Selection against carrier parents (strategy 1) and selection and mate allocation to avoid homozygous offspring (strategy 2)

### Scenario A

The impact of embryonic lethality on total profit was minor when only seven essential loci with LOF alleles were simulated. Therefore, there was little impact of the alternative mating strategies on this scenario as the loss per mating was only $1.40 in the absence of any selection or genotyping information. Breeding strategy 1 had a small impact on genetic gain in this scenario. A weighting of 1 on LethalA gave the highest $P, i.e. $113.87, as it maximized genetic progress while reducing the number of affected calves. The group of selected sires had a slightly lower $MB value of $140.61 than the base run, and a GLS of 0.005, as sires with fewer lethal alleles were selected compared to those in the base run (Table 6). No improvement from genotyping part of the herd was seen for breeding strategy 1 until at least 70% of the herd was genotyped, and the resulting improvement was only an increase of $P by $0.70/mating (Table 7).

**Table 7:** Costs of embryonic lethality^a^ and savings from genotyping ($30/test) when selecting to avoid heterozygous parents (strategy 1) for three scenarios^b^

Level of genotyping	% Herd genotyping	Dollars saved with reduction in occurrence of homozygous individuals (*aa*)					
		Before genotyping costs			After genotyping costs		
		A^b^	B	C	A	B	C
Base	0	$(140.00)	$(2490.43)	$(3043.75)	*$* ***(*** *140.00* ***)***	$(2490.43)	$(3043.75)
Low	10	$0.00	$669.18	$560.00	$(440.00)	$369.18	$260.00
	20	$0.00	$1004.18	$1831.30	$(740.00)	*$404.18*	*$1231.25*
	30	$0.00	$1273.56	$1833.80	$(1040.00)	$373.56	$933.75
Intermediate	40	$0.00	$1448.56	$2083.80	$(1340.00)	$248.56	$883.75
	50	$0.00	$1480.43	$2093.80	$(1640.00)	$(19.57)	$593.75
	60	$0.00	$1575.43	$2093.80	$(1940.00)	$(224.57)	$293.75
High	70	$70.00	$1677.93	$2093.80	$(2030.00)	$(422.07)	$(6.25)
	80	$70.00	$1688.56	$2173.80	$(2330.00)	$(711.45)	$(226.25)
	90	$70.00	$1755.43	$2173.80	$(2630.00)	$(944.57)	$(526.25)
	100	$70.00	$1767.93	$2193.80	$(2930.00)	$(1232.07)	$(806.25)

In italic characters = percentage of herd genotyped that maximized net profit for that scenario

^a^Per 100 matings

^b^Scenario A (7 loci with high-frequency LOF alleles), scenario B (76 loci with low-frequency LOF alleles), and scenario C (50 with high- and low-frequency loci)

(), numbers in parentheses indicate negative values

**Fig. 3:**
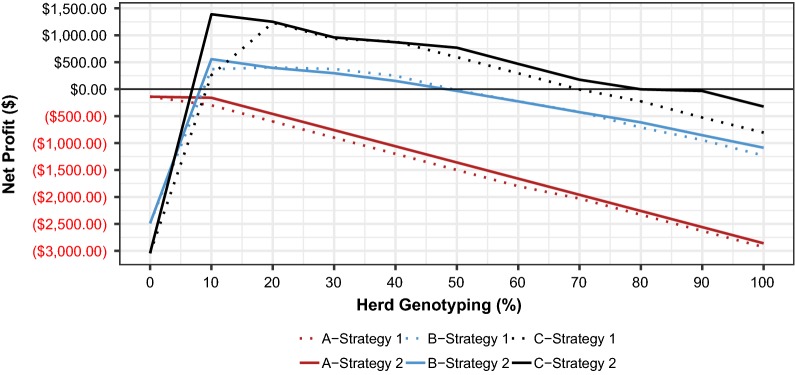
Net profit^a^ for different herd genotyping percentages ($30/test) for three scenarios^b^ given two breeding strategies^c^. ^a^Per 100 matings. ^b^Scenario A (7 loci with high-frequency LOF alleles), scenario B (76 loci with low-frequency LOF alleles), and scenario C (50 with high- and low-frequency loci). ^c^Strategy 1: selection against carrier parents (*Aa*); strategy 2: mate selection to avoid homozygous offspring (*aa*). (), numbers in parentheses indicate negative values

**Fig. 4:**
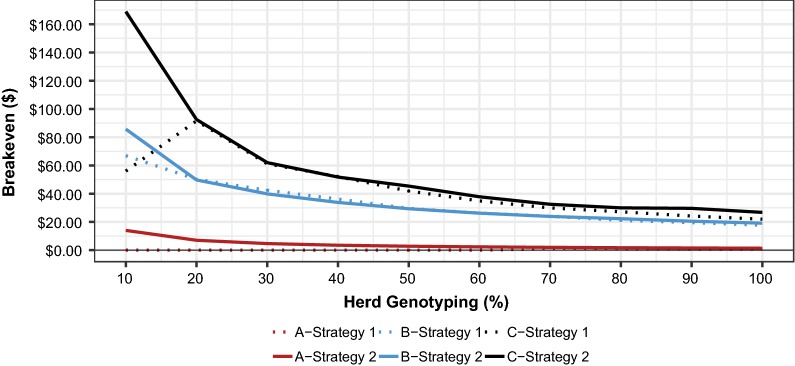
Breakeven ($/test) value of different herd genotyping percentages for three scenarios^a^ when using two breeding strategies^b^. ^a^Scenario A (7 loci with high-frequency LOF alleles), scenario B (76 loci with low-frequency LOF alleles), and scenario C (50 with high- and low-frequency loci). ^b^Strategy 1: Selection against carrier parents (*Aa*); strategy 2: Mate selection to avoid homozygous offspring (*aa*)

Any percentage of herd genotyped at $30/test decreased net profit (Fig. 3), and the breakeven genotyping cost never rose above $1/test for breeding strategy 1 in this scenario (Fig. 4).

With breeding strategy 2, the goal of maximizing $M and having no affected calves was achieved at the 0.001 weighting on LethalG (Fig. 2), which also maximized $P at the theoretical maximum $114.57 (Table 4). The group of selected sires at this weighting did not change relative to that of the base run; rather, mate allocation was used to avoid the occurrence of homozygous affected calves. Overall, breeding strategy 2 had a slight profit advantage ($114.57) compared to breeding strategy 1 ($113.87). Only 10% of the herd needed to be genotyped when using breeding strategy 2 to avoid all affected calves (Table 5), and the resulting improvement was a $140 savings (Table 8).

**Table 8:** Costs of embryonic lethality^a^ and savings from genotyping ($30/test) when selecting and allocating mates to avoid homozygous offspring (strategy 2) for three scenarios^b^

Level of genotyping	% Herd genotyping	Dollars saved with reduction in occurrence of homozygous individuals (*aa*)					
		Before genotyping costs			After genotyping costs		
		A^b^	B	C	A	B	C
Base	0	$(140.00)	$(2490.43)	$(3043.75)	$*(140.00)*	$(2490.43)	$(3043.75)
Low	10	$140.00	$857.06	$1689.50	$(160.00)	*$557.06*	*$1389.50*
	20	$140.00	$994.06	$1850.00	$(460.00)	$394.06	$1250.00
	30	$140.00	$1195.80	$1860.00	$(760.00)	$295.78	$960.00
Intermediate	40	$140.00	$1352.10	$2070.50	$(1060.00)	$152.13	$870.50
	50	$140.00	$1466.60	$2268.90	$(1360.00)	$(33.40)	$768.88
	60	$140.00	$1572.50	$2268.90	$(1660.00)	$(227.48)	$468.88
High	70	$140.00	$1672.60	$2275.00	$(1960.00)	$(427.39)	$175.00
	80	$140.00	$1783.60	$2396.90	$(2260.00)	$(616.43)	$(3.13)
	90	$140.00	$1845.20	$2665.80	$(2560.00)	$(854.81)	$(34.25)
	100	$140.00	$1913.00	$2677.30	$(2860.00)	$(1086.97)	$(322.75)

In italic characters = percentage of herd genotyped that maximized net profit for that scenario

^a^Per 100 matings

^b^Scenario A (7 loci with high-frequency LOF alleles), scenario B (76 loci with low-frequency LOF alleles), and scenario C (50 with high- and low-frequency loci)

(), numbers in parentheses indicate negative values

The value returned from testing never approached $30/test, so genotyping any percentage of the herd at this cost resulted in decreased net profit (Fig. 3). The breakeven value of genotyping 10% of the herd was $14. As the percentage of the herd genotyped increased, the value of genotyping decreased from $14 down to $1.40/test in the case of 100% of the herd genotyped. Collectively, these data suggest that if there are few embryonic losses occurring in the herd, as observed in scenario A, the value of genotyping to manage lethal recessive conditions is correspondingly very low, ranging from $0 to $14 (Fig. 4).

### Scenario B

This scenario modeled 76 essential loci with LOF alleles at low frequencies. In this case, the base scenario resulted in 12.45 lethal genotypes per 100 matings, which decreased $P by an average of $24.90 per mating in the absence of any selection or genotyping information (Table 4). A 0.1 weighting on LethalA for breeding strategy 1 maximized $P at $107.35 (Table 4), showing the best balance of maximizing $M while reducing the occurrence of affected calves. At this weighting, the $MB value of the group of selected sires was $140.75 and the average sire GLS was 0.265 (Table 6). Higher LethalA weightings reduced the GLS to 0 but were accompanied by a dramatic decrease in $MB of the selected sires, and an accompanying decrease in the average progeny index (Fig. 2).

Using this optimal LethalA weighting, low percentages of herd genotyped (10 to 30%) increased $P by $6.69 to $12.73 (Table 4), because a reduction in homozygous offspring was achieved (Table 5), resulting in savings of $669.18–$1273.56 before genotyping costs (Table 7). Using a $30/test value, net profit was optimized at 20% of the herd genotyped (Fig. 3). Beyond that, the marginal benefit of the test information was outweighed by the additional cost of genotyping and dropped below $30 when 50% of the herd was genotyped. Breakeven genotyping costs decreased as more of the herd was genotyped, i.e. it ranged from $66.92 at 10% down to $17.68 at 100% of the herd genotyped (Table 9, Fig. 4).

**Table 9:** Genotyping breakeven values for different percentages of herd genotyped in three scenarios^a^ given two selection strategies^b^

Level of genotyping	% Herd genotyping	Breakeven values					
		Strategy 1			Strategy 2		
		A	B	C	A	B	C
Low	10	$0.00	$66.92	$56.00	$14.00	$85.71	$168.95
	20	$0.00	$50.21	$91.56	$7.00	$49.70	$92.50
	30	$0.00	$42.45	$61.13	$4.67	$39.86	$62.00
Intermediate	40	$0.00	$36.21	$52.09	$3.50	$33.80	$51.76
	50	$0.00	$29.61	$41.88	$2.80	$29.33	$45.38
	60	$0.00	$26.26	$34.90	$2.33	$26.21	$37.81
High	70	$1.00	$23.97	$29.91	$2.00	$23.89	$32.50
	80	$0.88	$21.11	$27.17	$1.75	$22.29	$29.96
	90	$0.78	$19.50	$24.15	$1.56	$20.50	$29.62
	100	$0.70	$17.68	$21.94	$1.40	$19.13	$26.77

^a^Scenario A (7 loci with high-frequency LOF alleles), scenario B (76 loci with low-frequency LOF alleles), and scenario C (50 with high- and low-frequency loci)

^b^Selection against carrier parents (strategy 1) and selection and mate allocation to avoid homozygous offspring (strategy 2)

Profit per mating ($P) for breeding strategy 2 was again maximized ($108.79) when the lowest weighting (0.001) was placed on LethalG, but it was still $5.78 below the theoretical maximum since some embryonic mortality still occurred. At this weighting, $MB value of the group of selected sires was unchanged from that of the base run, but the average sire GLS decreased slightly to 0.284 (Table 6). The net savings in reduced embryonic lethality (Table 8), combined with the costs associated with genotyping 10% of the herd, resulted in the highest net profit (Fig. 3). Net profit for this breeding strategy was actually slightly lower than that for breeding strategy 1 for percentages of the herd genotyped ranging from 20 to 70% (Table 4, Fig. 3). Breakeven genotyping costs for breeding strategy 2 in this scenario ranged from $85.71 (10%) to $19.13 (100%) (Table 9, Fig. 4).

### Scenario C

This scenario modeled 50 essential loci with a random assortment of low and high LOF allele frequencies. In this case, the base run resulted in 15.22 lethal genotypes per 100 matings i.e. the highest value, which decreased $P by an average of $30.43 per mating. As such, the total opportunity cost of embryonic mortality per 100 matings was $3043.75. For breeding strategy 1, a weighting of 1 on LethalA maximized profit value ($99.48). The group of selected sires at this weighting had average $MB and GLS values of $133.75 and 0.167, as compared to $141.03 and 0.333 at the base run, respectively (Table 6). The profit impact of genotyping was greatest for this scenario (Fig. 3). Low percentages of the herd genotyped, i.e. 10 to 30%, increased $P by $3.77 at 10% and $13.40 at 30% of the herd genotyped (Table 4). With a reduction in the number of embryonic mortalities from 12.42 to 6.05 (Table 5), dollar savings before genotyping costs for low percentages of the herd genotyped (10–30%) ranged from $560.00 to $1833.80 (Table 7). Net profit was maximized at 20% of the herd genotyped with a value of $1231.25 (Table 7). Breakeven costs for genetic testing ranged from $91.56 at 20% to $21.94 at 100% of the herd genotyped (Table 9, Fig. 4).

A slight weighting of 0.1 on LethalG in breeding strategy 2 maximized $P at $110.31, which is $10.83 better than the $P resulting from breeding strategy 1 (Table 4). The group of selected sires had $MB and GLS values of $138.81 and 0.257, respectively (Table 6). The net savings in reduced embryonic lethality combined with the costs associated with genotyping 10% of the herd resulted in the highest net profit from genotyping (Table 8). When 40% of the herd was genotyped at $30/test, net profit for breeding strategy 2 was less than that associated with breeding strategy 1. For all other percentages of herd genotyped, selection to avoid homozygous offspring was more profitable than selection against carrier parents (Fig. 3). Breakeven genotyping costs were highest for breeding strategy 2 and scenario C, ranging from $168.95 (10%) to $26.77 (100% of the herd genotyped) (Table 9, Fig. 4). In this scenario, genotyping using a cost of $30/test was generally profitable at low to intermediate percentages of herd genotyped due to the relatively high number of embryonic losses that occurred in the absence of genotypic information.

## Discussion

Traditionally, calculations of a selection index do not incorporate the effect of specific alleles that result in embryonic or fetal mortality, since most selection indices assume additive relationships between genotypes, and trait values have a linear relationship with profitability [60]. The management of recessive lethal conditions became an important factor in cattle breeding in the 1950s because of dwarfism occurring in some breeds [61], and more recently because of several additional genetic defects that have occurred in popular beef seedstock pedigrees [62]. For many of these defects, DNA tests have been developed to identify individuals that carry the causal recessive lethal allele [62].

Almost a decade ago, Charlier et al. [63] suggested that using high-density SNP panels to accelerate the identification of certain mutations that cause defects within livestock populations would allow for immediate management within breeding practices. One of the important considerations in managing recessive alleles is the frequency of the recessive allele in the population. A number of different alleles that affect fertility have been identified in both beef and dairy cattle. Cole et al. [8] and Georges et al. [5] reviewed the recent studies on recessive haplotypes and recessive variants identified by whole-genome sequencing (reverse genetic screening) in cattle (Table 1).

In dairy cattle, four loci associated with embryo mortality, and three strong candidate causal mutations, were identified in Holstein, Montbéliarde, and Normande breeds [24]. A study of 337 Holstein–Friesian cows found three possible recessive lethal alleles in *selectin* genes that are required for embryo implantation and placental development [64]. However, more than 400 candidate LOF alleles were identified by whole-genome sequencing in a study of 6300 Belgian beef and 35,000 New Zealand dairy cattle. Testing 200 candidate offspring from carrier sire by carrier dam matings identified nine mutations that resulted in significant depletion of homozygotes, providing evidence that these alleles were true LOF variants [7]. Hoff et al. [6] identified seven loci with haplotypes that were not found in the homozygous state in Angus beef cattle, which suggests that these loci are possible candidates for LOF alleles. Recently, Jenko et al. [37] reported three haplotypes that carry putatively recessive lethal alleles in Aberdeen Angus, Charolais, and Simmental at population frequencies of 15.2, 14.4, and 8.8%, respectively. These studies suggest that LOF alleles, which impact fertility, are present in many cattle populations and are likely to be identified on an ongoing basis. As the number of identified mutations increases, animals not carrying LOF alleles will become increasingly rare [5].

In the absence of an estimate of the actual number of loci that are currently affected by LOF alleles, the value and optimum number of animals to genotype within a herd is difficult to predict. In this study, two breeding strategies were evaluated using scenarios in which the number of essential loci ranged from 7 to 76, and the mean LOF minor allele frequencies ranged from 0.0112 to 0.0847 (Table 2), which are representative of the values found in the literature (Table 1). With a full knowledge of the carrier status of each individual, the most profitable short-term breeding strategy was always strategy 2, i.e. simultaneous selection and mate allocation to avoid homozygous affected calves (*aa*) (which avoids carrier matings) compared to breeding strategy 1, i.e. indiscriminate selection against carrier parents (*Aa*) within a population. Mate allocation allowed for the matching of genetically superior carrier individuals, provided they had LOF alleles at different essential loci, and optimized the use of those individuals. Strategy 2 generally requires ongoing efforts to maintain benefits in subsequent generations, whereas the long-term benefits of strategy 1 will be cumulative over time and thus were not clearly reflected in this single generation simulation.

Other authors have explored how to manage recessive LOF alleles. Cole [38] modeled constraining inbreeding based on genomics [65] and added in the model an economic penalty for Mendelian disorders, which ranged from $20 to $200 for hypothetical recessive loci. This approach reduced LOF allele frequencies and was especially useful for low-frequency LOF with small economic value. This simulation did not allow for the management of parental coancestry as was modeled in our study using MateSel. The need for MateSel to access pedigree and index information on all candidates complicates the ease-of-implementation and as posited by Cole, “sometimes it is better to have an imperfect mate allocation tool, than no tool at all”. Currently, most beef producers are culling carriers and this is likely to become infeasible as more LOF mutations are discovered. As more genomic information becomes available due to increased rates of genotyping, it is likely that software to maximize the use of this information for optimal mate selection and allocation will become increasingly valuable for breed associations, and their members.

In this study, we modeled just a single round of mating in order to reflect the real-life situation of tactical decision-making. However, we managed long-term inbreeding/diversity by setting a limit on parental coancestry as is commonly done in practice. Moreover, our LethalA metric is essentially an additive criterion that aims at generations beyond the progeny generation. Further studies using multiple generations would be useful to uncover the long-term impact on genetic gain and embryonic losses of the mate selection and allocation strategies that were examined in this study over many generations.

Genotyping information enabled the management of LOF alleles. However, the breakeven value derived from genotyping depended highly on the scenario and mating strategy modeled. In scenario A, with only seven LOF alleles, the breakeven value of genotyping was never more than $1 with breeding strategy 1 and $2 with breeding strategy 2. In contrast, in scenario C, with 50 LOF alleles of variable frequencies, it could reach a breakeven value as high as $168.95/test when using simultaneous mating and selection to avoid homozygous affected calves (*aa*) and genotyping only 10% of the herd. Clearly, the breakeven value of genotyping information increases in proportion to the number of embryonic losses that are anticipated in the absence of genotyping information. In the future, it is likely that the identification of recessive conditions will be part of routine genotyping, and so there will be no separate genotyping costs for LOF alleles. The availability of genome-wide data for genomic selection may allow for the imputation of LOF alleles at essentially no additional cost, but with an accuracy lower than 100%. The use of genotyping for multiple purposes, including the tantalizing prospect of genomic mate selection to concentrate the most favorable complementary alleles into offspring [5], will likely make it cost-effective for considering and managing all known LOF alleles, irrespective of frequency, in mate selection and allocation decisions.

Genotyping a larger percentage of the herd provided more information to decrease embryonic mortalities compared to genotyping smaller percentages of the herd, although this depended on the number of loci and allele frequencies. Within each scenario and breeding strategy, $P was maximized when 70 to 100% of the herd was genotyped; however, there was little additional value generated by increased levels of genotyping to offset the additional costs. Breakeven genotyping costs typically decreased as the percentage of the herd genotyped increased in the different scenarios. The exception to this was for scenario C for which the marginal value of genotyping increased as the percentage of the herd genotyped increased from 10 to 20% when using breeding strategy 1 due to the added value derived from that additional information to avoid the use of carrier parents (Fig. 4). In all other cases, the marginal value of additional dollars saved by increasing the proportion of the herd genotyped decreased; therefore, the breakeven genotyping costs decreased as the percentage of herd genotyped increased.

If scenario A approximates the true frequencies, as was suggested by studies reporting seven to nine lethal LOF mutations [6, 7], then the actual value of genotypic information to manage recessive lethal conditions may be low, at least in the short term. However, with just seven LOF loci, there may be some prospect to eliminate the LOF alleles, and therefore some benefit to long-term management. In the other two simulations with more than 50 loci with LOF alleles, genotyping a small percentage of the herd improved net profit. Figure 4 that illustrates the breakeven costs of genetic testing is perhaps the most illustrative of the complexities of the value of a genetic test, since it shows the impact that the percentage of herd genotyped, breeding strategy, and different numbers of essential loci and LOF frequencies can have on the value of genotyping information. These values reveal the importance of the underlying scenario in developing estimates of the economic value of genotyping information for managing recessive lethal conditions.

Kinghorn [41] described genotyping strategies that used genotyping probabilities from segregation analysis to help choose which individuals and loci to genotype. It has been reported that genotyping sires, then dams, and then non-parents, added performance to a population with a low allele frequency. Undoubtedly, these are more strategic approaches than those modeled in our study, and additional tools, such as Geneprob [57], can be used to estimate the probability that an animal is a carrier, as was done in our study. The choice of which individuals to genotype should likely be based on the influence of each individual in the breeding program (e.g., bulls > cows), and/or its estimated breeding value. Selection of influential individuals for genotyping should be done in such a way that it contributes useful information to the whole population, e.g. by determining the carrier status of key influential sires [41].

In the Australian Angus population, DNA test results and pedigree information were used to calculate the probability that a non-tested animal was a carrier of the arthrogryposis multiplex (AM) syndrome with the software program Geneprob [57, 66]. These results were analyzed and are publicly available on the Australian Angus Association’s website. Along with this program, the association designed educational programs to help members understand how to identify and manage recessive lethal alleles. By implementing these practices, producers decreased their use of AM sires, thus decreasing the carrier individuals within the Australian Angus population [67]. This was similar to our breeding strategy 1 of selecting against carrier parents. Although genotyping animals decreased the use of carrier sires, it may not have been an optimum approach given the results of the current study. Managing genetic defects is ultimately a trade-off between avoiding affected individuals in the short-term and eliminating LOF alleles in the long run [38].

Although this example shows that the reduction of recessive lethal alleles is possible, complete elimination of these alleles may be difficult [68]; thus, continued long-term management is necessary. Allen et al. [69] modeled the impact of a sire that carries a recessive lethal genetic condition. Random mating herds that use an occasional carrier sire had a low occurrence of homozygous affected calves (less than 1%). However, within a self-replacing herd, where carrier females may stay in the herd for around 20 years, consistent management is required to avoid recessive lethal conditions [69].

In our study, a value of $200 was used as the cost of a homozygous lethal progeny, and the economic factors influencing this value will vary. Factors such as the number of animals within the herd, whether the herd is self-replacing, and the degree of risk that producers are willing to accept should also be taken into consideration [70]. A limit of our study is that only a single herd and a single generation were modeled, thus it is not possible to draw any conclusions about the long-term dynamics of LOF in larger populations more generally. Interestingly, in a simple simulation study that assigned a small ($20–$40) or large ($200–$400) economic value to a locus with a recessive LOF allele had little impact on decreasing the frequency of the deleterious allele over time [38]. In that example, increasing the economic cost of recessive LOF alleles only was not sufficient to accelerate the rate at which undesirable alleles were eliminated from the population.

Mate selection to avoid affected progeny may help LOF alleles to survive in the population, if their frequency is sufficiently high such that random mating would otherwise have resulted in significant selection against them. At lower LOF allele frequencies, mate selection to avoid affected progeny may reduce the frequency of LOF alleles across many loci in the population, since individuals that carry no LOF alleles, few LOF alleles, and/or LOF alleles that are at a lower frequency in the population, will be easier to match with mates to lower the probability of progeny mortality. However, it seems prudent, in practice, to include some weighting to avoid the selection of carriers, as well as weighting to reduce the prevalence of affected progeny. The appropriate balance between short- and long-term management will also depend on the period under consideration, since it will take a long time to eliminate LOF alleles from the population if carriers are allowed to qualify as parents because they will continue to generate heterozygous carrier offspring.

As sequencing projects identify more essential loci and LOF alleles, breed associations will need to develop policies on the management of lethal recessive alleles. When considering the amount of emphasis to place on lethal recessive genetic conditions, decisions on the appropriate balance of short- or long-term management of LOF alleles should be made first. If short-term management is prioritized, essentially by decreasing the occurrence of affected calves (*aa*), the optimal solutions from the scenarios presented here suggest that a slight emphasis is sufficient for improved mate allocation to avoid LOF carrier matings at the same locus. However, if long-term management of LOF alleles is also considered as important, there would be value in decreasing the number of carrier animals within the population, and some value would need to be assigned to avoiding carrier parents to achieve this objective. While short-term profit might be maximized by strategy 2, some weighting should be given to long-term elimination of defects from the population using some weighting on strategy 1.

Future research will likely elucidate a more accurate representation of the approximate number of loci affected by LOF mutations and the frequencies at which they occur within cattle populations. Once this becomes clearer, optimal mating and genotyping strategies to maximize overall producer profit can be modeled, although it will be necessary to consider the appropriate balance between avoidance of carrier matings (i.e., short-term producer benefit) and eliminating defects (i.e., long-term industry benefit), which will likely vary depending on the stakeholder. It is likely that the management of a suite of recessive lethal conditions will require the use of mate allocation programs such as MateSel to incorporate LOF information into mate selection decisions.

## Conclusions

The most profitable short-term breeding strategy given a perfect knowledge on LOF genotypes was simultaneous selection and mate allocation to avoid the potential for producing homozygous affected offspring compared to indiscriminate selection against carrier parents in the simulations modeled in our study. Before accounting for genotyping costs, $P increased within each simulation and breeding strategy as the percentage of the herd genotyped increased. However, genotyping 100% of the herd did not result in the maximum net profit when accounting for genotyping costs. Genotyping some percentage of the herd tended to show the greatest net profit increase in scenarios where a large number (≥ 50) of loci were associated with LOF alleles. Genotyping information does enable better management of lethal recessive alleles; however, the value of that information must be weighed carefully against the associated genotyping costs. As more LOF alleles are identified, it is likely that some genotyping information combined with mate selection software will be required to correctly manage this information and optimize mate selection and allocation to simultaneously increase genetic gain, control inbreeding, minimize recessive lethal matings, and maximize net profit from breeding decisions.

## Data Availability

MateSel is available free for research via matesel.une.edu.au, and available for commercial use under licence via www.matesel.com.
